# Large myoma receiving multiple collateral primary parasitic blood supply

**DOI:** 10.1002/ccr3.5945

**Published:** 2022-06-09

**Authors:** Nikolaos Kathopoulis, Konstantinos Kypriotis, Michail Diakosavvas, Ioannis Chatzipapas, Dimitrios Zacharakis, Themistoklis Grigoriadis, Athanasios Protopapas

**Affiliations:** ^1^ Endoscopic Surgery Unit 1st Department of Obstetrics & Gynecology National and Kapodistrian University of Athens, “Alexandra” Hospital Athens Greece

**Keywords:** laparoscopy, myomectomy, parasitic myoma

## Abstract

We describe a rare case of a pedunculated myoma receiving multiple de‐novo developed parasitic collateral blood supply from the adjacent organs. The main feeding vessels arise from the omentum and the bladder.

## Abstract 2

Primary parasitic blood supply on myomas is a rare pathological entity that make laparoscopic myomectomy a demanding operation.

A 30‐year‐old patient presented for abnormal uterine bleeding secondary to fibromas. A large pedunculated myoma was observed receiving blood supply from three sites other than the pedicle during laparoscopy (Figure 1A). The first collateral circulation came from the bladder through multiple markedly dilated vessels (Figure 1B). Another feeding vessel cluster originated from the lateral peritoneum below the external iliac vein (Figure 2). The main blood supply came from the omentum through a complex of long, large‐diameter feeding vessels (Figure 3). Bipolar diathermy was applied to ligate the feeding vessels safely, and all six fibromas were removed (Figure 4).

**FIGURE 1:**
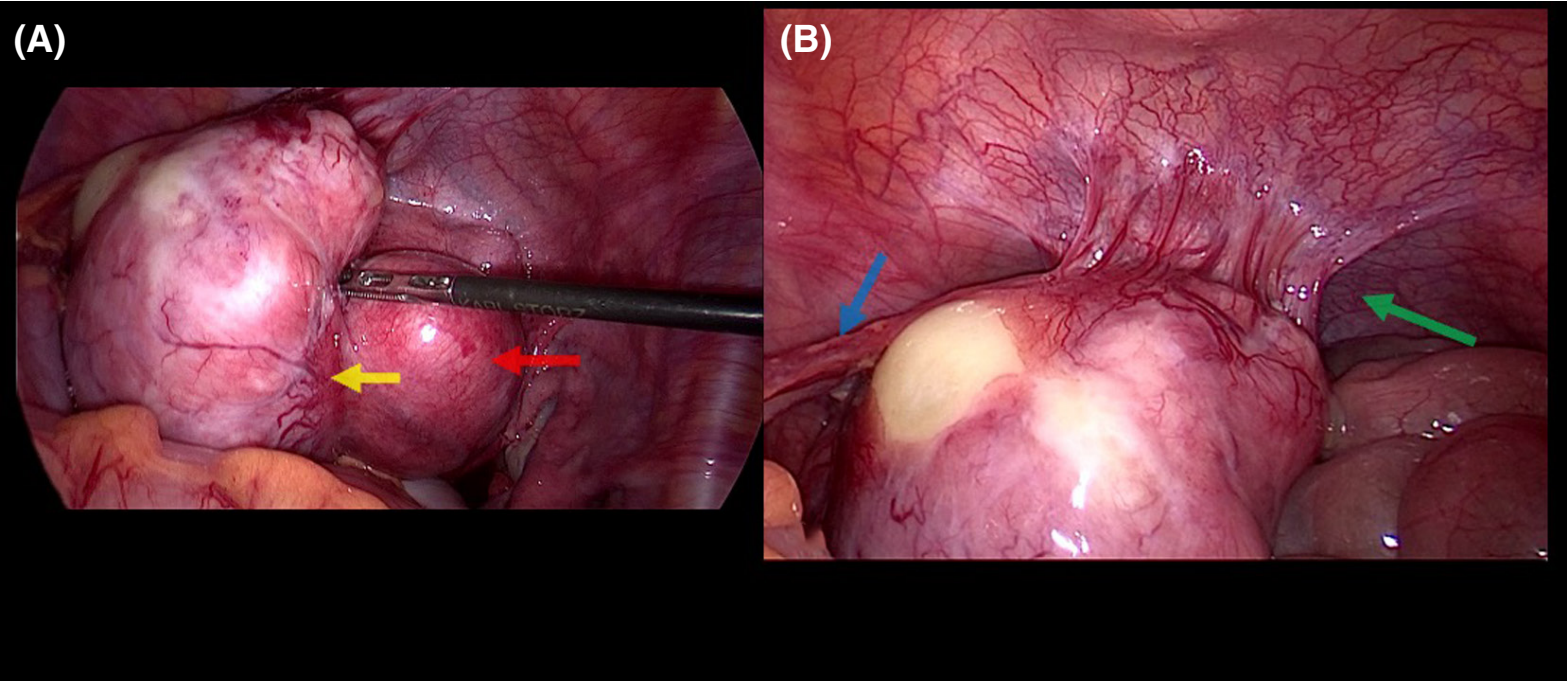
(A) Myoma pedicle (yellow arrow) attached to the serosal over another fundal myoma (red arrow). (B) First collateral blood supply originating from the bladder containing multiple vessels (green arrow). Second parasitic blood supply from the omentum (blue arrow)

**FIGURE 2:**
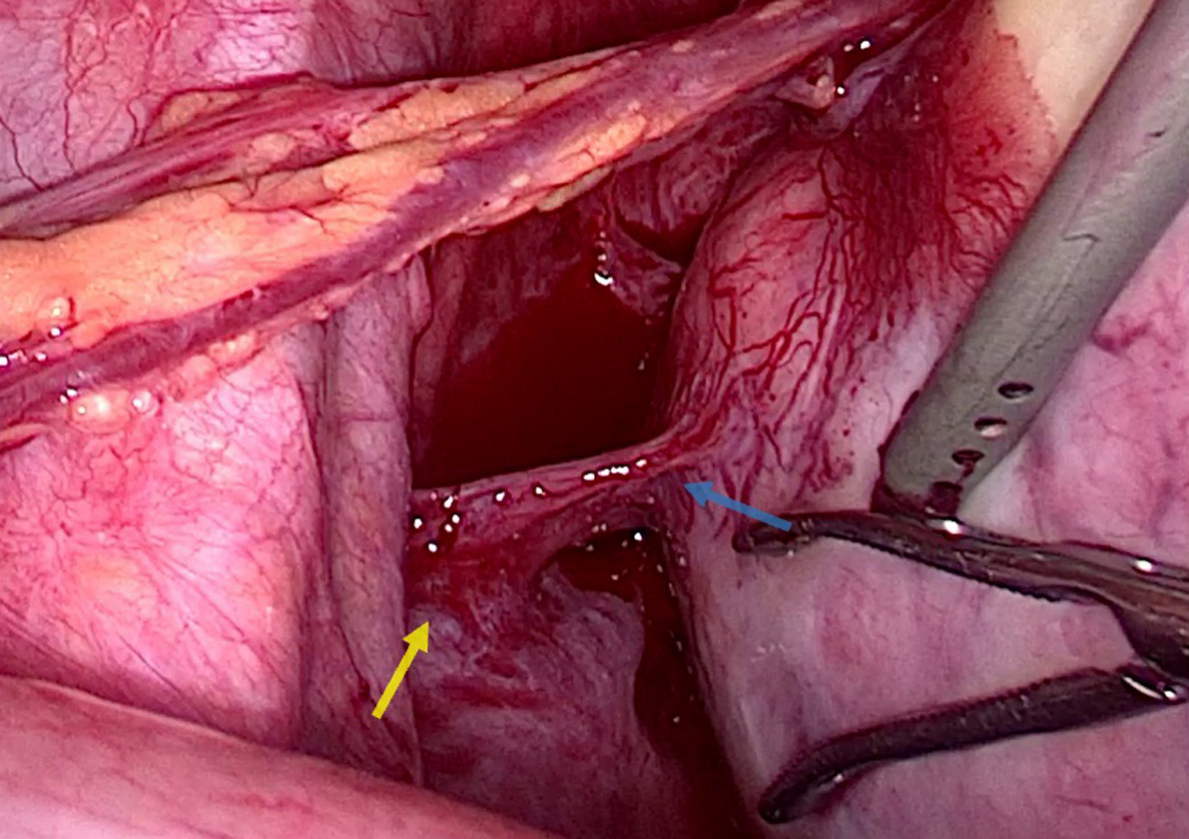
Feeding vessel cluster (blue arrow) originating from the lateral peritoneum below the external iliac vein (yellow arrow)

**FIGURE 3:**
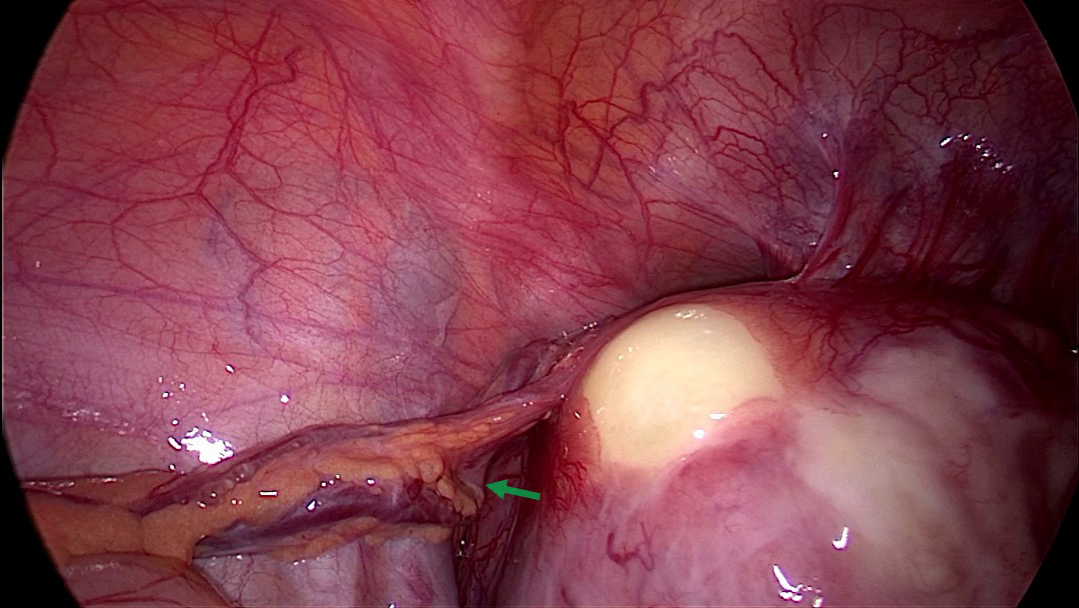
Main blood supply of the myoma from the omentum through a small number of long, large‐diameter feeding vessels (green arrow)

**FIGURE 4:**
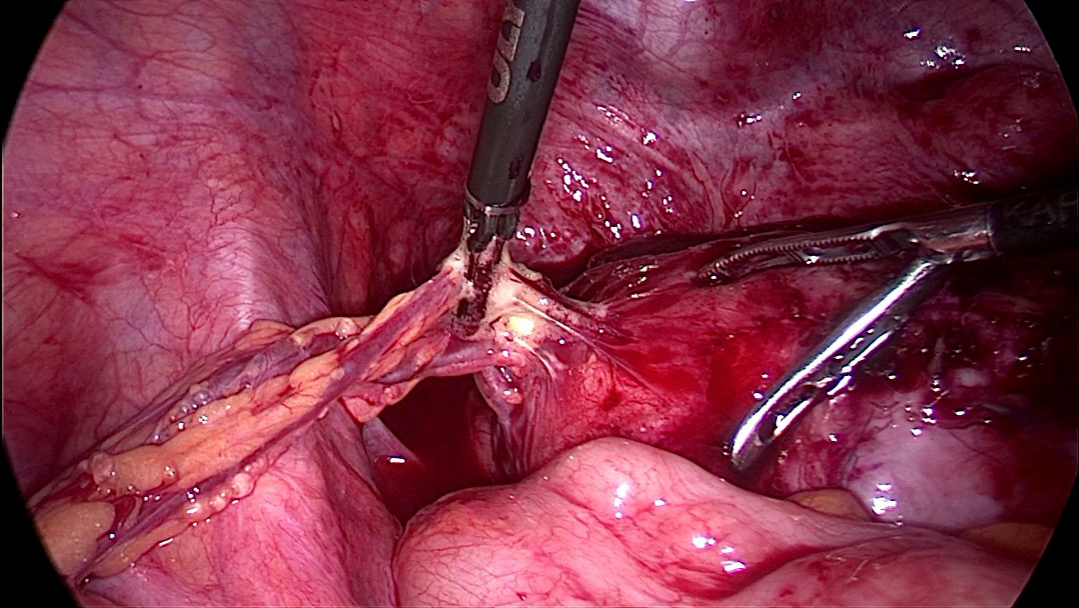
Bipolar energy was used to ligate safely the collateral blood supply

Parasitic myomas are a rare variant of pedunculated myomas that have overgrown their uterine blood supply and separated from the uterus.^1^ The majority are iatrogenic parasitic myomas that the seeding of remnants may cause after using power morcellation during a previous laparoscopic myomectomy.^2^ The uniqueness of the presented case is the primary collateral circulation of the myoma, with absence of previous surgery that would explain its presence as part of the adhesion formation process. In most cases reported, the parasitic blood supply comes from one site; in this case, though, three different sites provide blood perfusion to the myoma.

## 1 QUESTION

Are parasitic myomas a pathology secondary only to myomectomy?

## AUTHOR CONTRIBUTIONS

NK involved in conception and design, and served as a responsible surgeon. KK served as a responsible surgeon and wrote the manuscript. MD served as a responsible surgeon. IC collected and created the figures. DZ collected and created the figures. TG and AP designed the project and edited the manuscript. All authors have read and approved the final manuscript.

## CONFLICT OF INTEREST

The authors declare no conflict of interest.

## CONSENT

The authors declare that a written informed consent was obtained from the patient for publication of this case report. No patient identifying data has been released in the article.

## Data Availability

Data are available upon request to the corresponding author.
